# WALANT Scaphoid Fixation Under Field Sterility: A Case Report and Description of Technique

**DOI:** 10.1177/22925503231213866

**Published:** 2023-11-20

**Authors:** Brett Ponich, Madison Turk, Maleka Ramji, Aaron Knox

**Affiliations:** 1 Division of Plastic and Reconstructive Surgery, 2129 University of Calgary, Calgary, AB, Canada; 2 Cummings School of Medicine, 2129 University of Calgary, Calgary, AB, Canada; 3 2129 University of Calgary, Calgary, AB, Canada

**Keywords:** scaphoid, WALANT, field sterility, champ stérile, scaphoïde, WALANT

## Abstract

Wide Awake Local Anesthesia No Tourniquet (WALANT) technique has become an increasingly popular method of treating common hand pathologies. We present its application in the treatment of an acute minimally displaced scaphoid fracture. In addition, we demonstrate this can be achieved with field sterility using minimal surgical PPE and draping in a minor surgery setting, outside the main operating room theatre.

## Résumé

La technique de WALANT (*Wide Awake Local Anesthesia No Tourniquet*, ou anesthésie locale sur patient éveillé sans usage du garrot) est de plus en plus populaire pour traiter des pathologies courantes de la main. Les auteurs en présentent les applications pour le traitement d’une fracture aiguë du scaphoïde à peine déplacée. Ils démontrent également qu’ils peuvent l’effectuer sous champ stérile à l’aide d’équipement de protection individuelle chirurgical et de champ opératoire minimaux, hors du bloc opératoire.

## Background

Scaphoid bone fractures are one of the most common carpal fractures presenting to hand surgeons.^1^ While conservative management is considered appropriate for stable fractures, unstable or minimally displaced fractures are often treated in the main operating theatre with closed, possible open reduction and internal fixation.^1‐3^ Wide Awake Local Anesthesia No Tourniquet (WALANT) surgery has become increasingly popular and has been linked to shorter operative procedure times, decreased patient recovery time, and overall health system savings.^4‐6^ Furthermore, post-surgical infection rates from this technique have been shown to be comparable to operating in main operating theatre under sterile conditions.^7^ We report our technique of percutaneous internal fixation of a minimally displaced scaphoid waist fracture using a headless compression screw. The procedure was performed in a minor surgery setting without an anesthetist using WALANT technique. Field sterility using minimal surgical PPE and sterile drapes was employed. We describe specific technique details to allow our approach to be reproducible with success by other hand and wrist surgeons.

## Methods

The patient's history and investigations were collected through a review of electronic medical records. The patient granted consent for photographic documentation and public presentation within the article.

**Figure 1.**
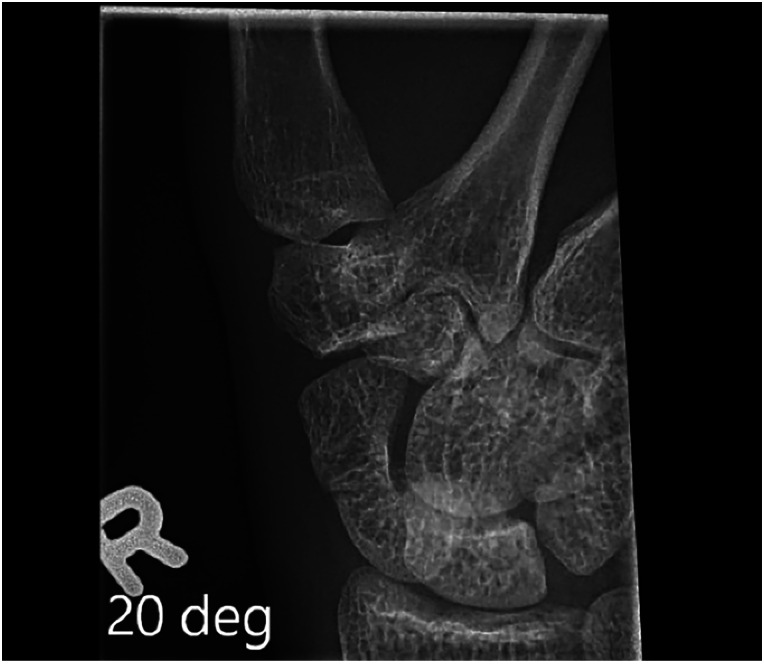
Mid-waist scaphoid fracture.

We report on a 21-year-old, right-hand-dominant male working part-time as a construction framer. He sustained a fall on outstretched hand injury while in his home. Post-injury radiographs confirmed a right scaphoid waist fracture with minimal comminution and approximately 1 mm displacement (Figure 1). He was referred to plastic surgery for consultation. A focused hand and wrist examination revealed right wrist swelling and ecchymosis with tenderness over the snuffbox and scaphoid tubercle. The decision to fixate was made due to patient preference as well as the location and displacement of his fracture.

## Operative Technique

The patient was placed on the operating bed with the operative arm extended on an arm board. We began with the administration of 20 mL of 1% Lidocaine with Epinephrine to create a full wrist block of the median, ulnar, radial, anterior, and posterior interosseous nerves, with additional infiltration over the volar thenar eminence.

Local anesthetic was infiltrated subcutaneously proximal to the wrist crease and extended circumferentially around the wrist field block fashion. An ulnar nerve-specific block was then achieved by inserting the needle on the volar-ulnar aspect of the wrist deep to the flexor carpi ulnaris and 1-2 cc's were administered. Direct median nerve block was performed by then inserting the needle centrally in the volar aspect of the palm just proximal to the carpal tunnel and injecting 1 cc into the carpal tunnel space. The DRUJ was palpated and the needle was advanced proximal to this through the interosseous membrane. Local was administered while withdrawing the needle to anesthetize the anterior and posterior interosseous nerves. To ensure adequate anesthetic, an additional 2-3 cc was injected in the thenar eminence, volar and superficial to the scaphoid.

**Figure 2.**
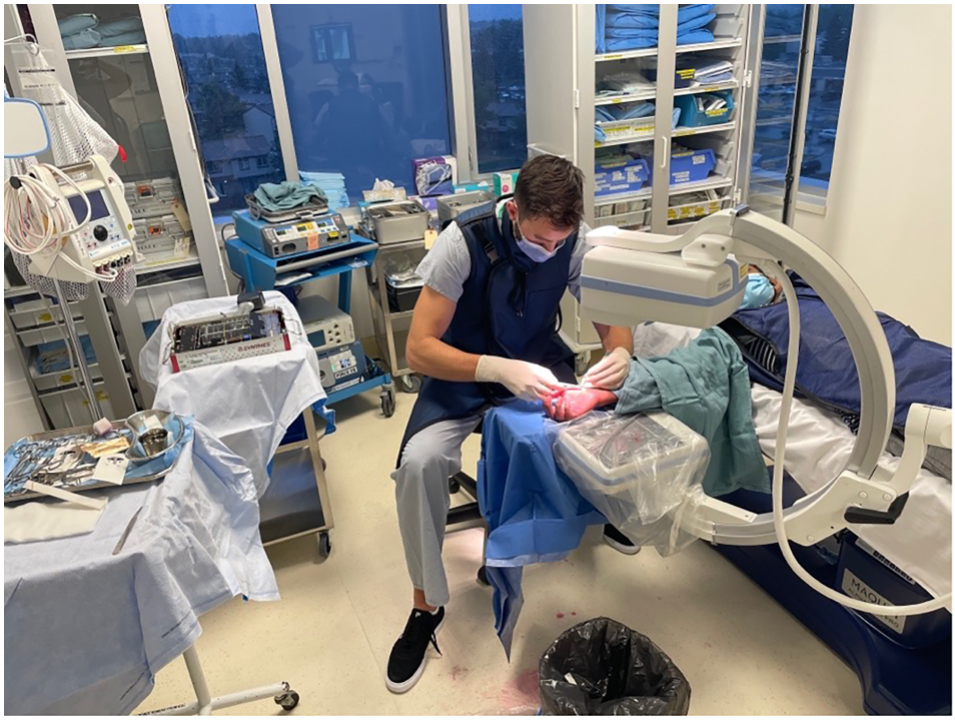
Equipment position and surgical attire.

**Figure 3.**
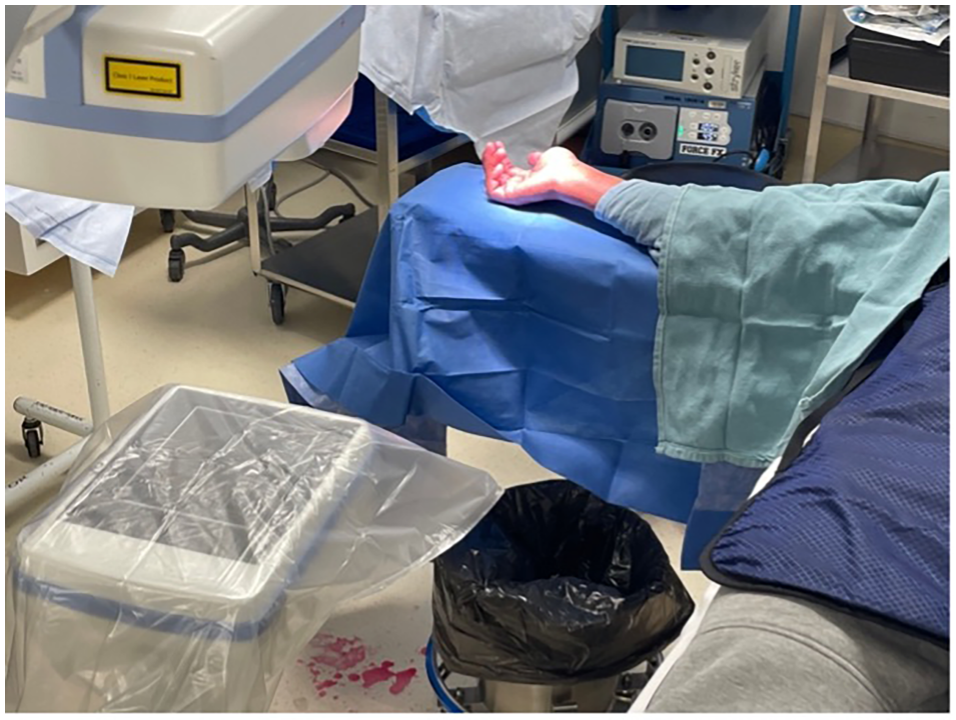
Draping of arm and fluoroscopy.

**Figure 4.**
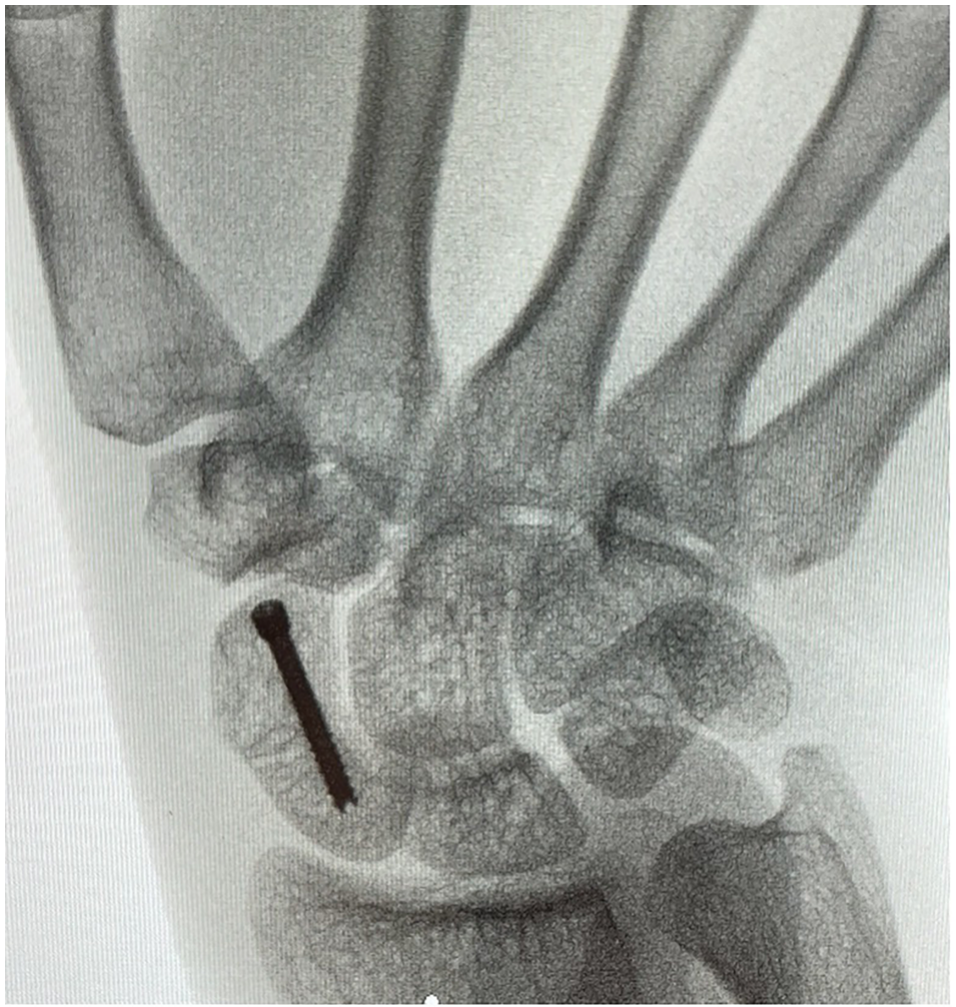
Anterior–posterior X-ray post screw placement.

**Figure 5.**
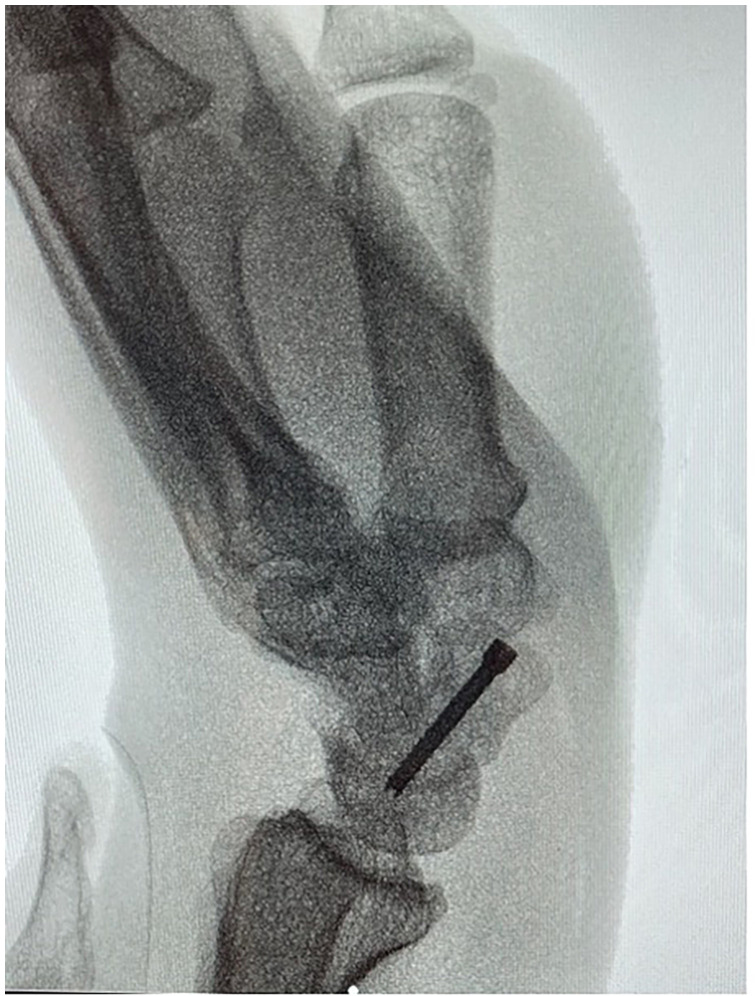
Lateral X-ray post screw placement.

The anesthetic was left to take effect while the equipment for the procedure was prepared, and the prepping and draping took place. Approximately 5 min transpired between the initial injection and the beginning of the procedure. The patient's extremity was prepped from fingers to mid forearm using chlorhexidine and wrapped with two standard sterile towels. The surgeon and assist wore generic non-sterile surgical scrubs and sterile gloves (Figure 2). The mini fluoroscopy image intensifier was draped with a bowel bag for sterility (Figure 3). A minor surgery plastics tray was opened, along with a single gown pack. The mini c-arm was then used to mark appropriate landmarks for the volar approach. The initial position of a k-wire was made by a percutaneous puncture at the base of the thumb carpometacarpal joint and advanced to the distal pole of the scaphoid. Two k- wires were placed, one for screw advancement and the other for rotational stability. Appropriate screw length was measured and a 2.4 mm Synthes self-drilling self-tapping headless compression screw was placed along the guide wire. Final images were confirmed with mini fluoroscopy (Figures 4 and 5). The skin was closed with a single 5-0 chromic suture and a removable splint was applied. The procedure was completed in less than 15 min.

**Figure 6.**
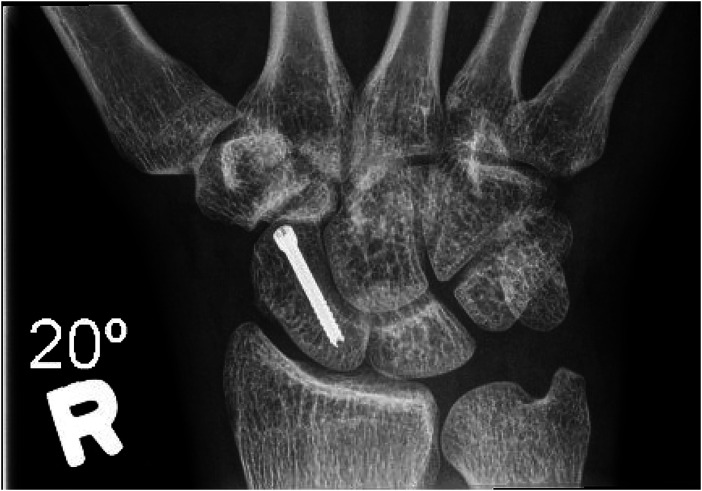
8-week follow-up X-ray.

The patient remained awake and pain-free for the duration of the procedure. Due to this, he was able to be made aware of each step, allowing him intimate knowledge of the fixation. Post-operative care and follow-up instructions were given concurrently during the procedure and explained in the context of each step, furthering his understanding of the rationale behind the instructions. He was then able to leave without any post-anesthetic observation period, upon completion of the surgery. After surgery, he was referred to hand therapy for a thermoplastic splint which he will wear continuously for 8 weeks. The patient was then followed up at 8 weeks for repeat X-ray (Figure 6), and 3 months. The fracture was clinically determined to be healed at this time.

## Results

The patient was seen at 8 weeks and 3 months for follow-up. On exam, he was non-tender, had full wrist and thumb range of motion, and had no signs or symptoms of infection. He was discharged from the clinic and informed to follow up as needed if concerns develop.

## Discussion

WALANT technique is an increasingly popular choice for hand surgeons managing a diverse array of traumatic hand injuries.^8,9^ This method has been previously described for scaphoid fracture operative management. Critical to success, is the effectiveness of the local block, ensuring the patient remains comfortable throughout the procedure. WALANT facilitates completion procedures outside of the main operating room theatre. As such, a reduced number of sterile materials and draping can be employed.

Surgeons may be hesitant to adopt this technique due to the perceived increase in infection risk. However, field sterility in conjunction with WALANT technique has been associated with low infection rates in upper extremity procedures.^7^

Reduction in the single-use draping equipment is both time and cost-saving. We demonstrate this approach is safe and our patient did not develop the potential complication of post-operative infection. Furthermore, the ability to communicate with the patient intra-operatively allows for more efficient use of time, as surgical steps and follow-up information were delivered concurrently with the procedure. In addition, room turnover time is reduced, allowing for greater efficiency and likely more cases completed.

## References

[1] Phillips TG Reibach AM Slomiany WP . Diagnosis and management of scaphoid fractures. Am Fam Physician. 2004;70(5):879‐884. Accessed September 2, 2022. https://www.aafp.org/pubs/afp/issues/2004/0901/p879.html 15368727

[2] Brogan DM Moran SL Shin AY . Outcomes of open reduction and internal fixation of acute proximal pole scaphoid fractures. Hand (N Y). 2015;10(2):227‐232. doi:10.1007/S11552-014-9689-8 26034435 PMC4447653

[3] Rhemrev SJ Ootes D Beeres FJP Meylaerts SAG Schipper IB . Current methods of diagnosis and treatment of scaphoid fractures. Int J Emerg Med. 2011;4(1):4. doi:10.1186/1865-1380-4-4 21408000 PMC3051891

[4] Chatterjee A McCarthy JE Montagne SA Leong K Kerrigan CL . A cost, profit, and efficiency analysis of performing carpal tunnel surgery in the operating room versus the clinic setting in the United States. Ann Plast Surg. 2011;66(3):245‐248. doi:10.1097/SAP.0B013E3181DB7784 21042185

[5] Codding JL Bhat SB Ilyas AM . An economic analysis of MAC versus WALANT: a trigger finger release surgery case study. Hand. 2017;12(4):348‐351. doi:10.1177/1558944716669693 28644939 PMC5484446

[6] Schank KJ Engwall AJ Kuhns BW Oakes TC Bray SM Clarkson JHW . Guidelines for wide-awake local anesthesia surgery with no tourniquet in the office setting using field preparation sterility. Plast Reconstr Surg. 2023;151(2):267e‐273e. doi:10.1097/PRS.0000000000009850 36696323

[7] Avoricani A Dar QA Levy KH Kurtzman JS Koehler SM . WALANT Hand and upper extremity procedures performed with minor field sterility are associated with low infection rates. Plast Surg. 2022;30(2):122‐129. doi:10.1177/22925503211003840 PMC9096863 35572084

[8] Kurtzman JS Etcheson JI Koehler SM . Wide-awake local anesthesia with no tourniquet: An updated review. Plast Reconstr Surg Glob Open. 2021;9(3):e3507. doi:10.1097/GOX.0000000000003507 PMC7997095 33786267

[9] Folberg CR Alves JAO Pereira FMS Pedrozo VB . WALANT Na osteossíntese percutânea do escafoide. Rev Bras Ortop. 2022;57(06):1070‐1073. Published online 2022. doi:10.1055/s-0041-1726070 PMC9757964 36540748

